# Association between Chronic Kidney Disease and Chronic Rhinosinusitis: A Longitudinal Follow-Up Study Using a National Health Screening Cohort

**DOI:** 10.3390/jpm14030268

**Published:** 2024-02-29

**Authors:** Heejin Kim, Tae Jun Kim, Mi Jung Kwon, Jee Hye Wee, Sung Kwang Hong, Hyo Geun Choi, Joong Seob Lee

**Affiliations:** 1 Department of Otorhinolaryngology-Head & Neck Surgery, Hallym University Sacred Heart Hospital, Anyang 14068, Republic of Korea; mir5020@hallym.or.kr (H.K.); weejh07@hallym.or.kr (J.H.W.); yerami@hallym.or.kr (S.K.H.); 2 Department of Medicine, Samsung Medical Center, Sungkyunkwan University School of Medicine, Seoul 06351, Republic of Korea; 3 Department of Pathology, Hallym University Sacred Heart Hospital, Anyang 14068, Republic of Korea; 4 Suseo Seoul ENT Clinics, Seoul 06349, Republic of Korea; pupen@naver.com; 5 MD Analytics, Seoul 06349, Republic of Korea

**Keywords:** chronic rhinosinusitis, chronic kidney disease, cohort study

## Abstract

Chronic kidney disease (CKD) is a leading cause of global mortality. While recent reports suggest potential connections between CKD and chronic rhinosinusitis (CRS), further research is needed to elucidate the direct association between CKD and CRS. This study investigated the association between CKD and CRS using data from the Korean National Health Insurance Service Health Screening Cohort. Participants were recruited according to medical claim codes, and individuals with CKD were matched in a 1:4 ratio with the control group. Covariates, such as demographics, health-related data, and medical history were used. The incidence rates and hazard ratio of CRS were analyzed. A further analysis was performed based on the presence of nasal polyps. Among the 514,866 participants, 16,644 patients with CKD and 66,576 matched controls were included in the analysis. The CKD group demonstrated a higher incidence of CRS than the controls: 18.30 versus 13.10 per 10,000 person-years. The CKD group demonstrated a higher risk of CRS than the control group (1.28 adjusted hazard ratio). In additional analyses, the CKD group did not exhibit a statistically significant correlation for the development of CRS with nasal polyps. This study suggests that CKD is associated with an increased risk for CRS.

## 1. Introduction

Chronic kidney disease (CKD) is a common disorder that is defined as renal function impairment, denoted by a glomerular filtration rate (GFR) below 60 mL/min per 1.73 m^2^ or indicators of kidney injury that persist for at least 3 months irrespective of the underlying cause [1]. In the adult population, the prevalence of CKD is approximately 15% in the US and 13% in Korea [2,3]. The count of patients with CKD and end-stage kidney disease is steadily increasing [4], and the progressive loss of kidney function is ultimately linked to renal replacement, such as dialysis and kidney transplantation. Above all, CKD has emerged as a leading cause of worldwide mortality [5,6].

Chronic rhinosinusitis (CRS) is characterized by persistent inflammation of the paranasal sinuses and nasal mucosa for at least 3 months [7]. CRS is a common disorder worldwide, with a prevalence of 14.2% in the US and 6.95% in Korea [8,9]. Major symptoms of CRS include nasal stuffiness, rhinorrhea, facial pain, and hyposmia [10]. Although these symptoms do not seem to be critical compared to other illnesses, patients with CRS have worse level of pain and social functioning compared to individuals with other chronic disorders, such as angina pectoris, congestive heart failure, and backache [11]. There are two subtypes of CRS based on phenotypic differences: CRS with nasal polyps (NPs) and CRS without NPs [12].

Recent studies have indicated a potential connection between CKD and sinonasal conditions, such as epistaxis, infections, and olfactory dysfunction [13,14,15]. However, only a few studies have assessed the direct association between CKD and CRS. A few case series on sinusitis and kidney disease have been reported [16,17]. The association between CKD and CRS has been unclear and remains an active research area.

This study examined the correlation between CRS and CKD in a population-based national cohort. We adjusted for sociodemographic factors, lifestyle factors, laboratory data, and medical history for an accurate analysis. We performed further analyses according to the presence of NPs, which compared the risk of CRS with and without NPs between CKD and control group. Although there are insufficient explanations for the link between CKD and CRS, these analyses may provide further insights into the underlying mechanisms.

## 2. Materials and Methods

### 2.1. Ethics

This study received approval from the ethics committee at Hallym University (2019-10-023). The Institutional Review Board waived the requirement for written informed consent. All analyses adhere to the guidelines and bylaws established by Hallym University’s ethics commission. A comprehensive overview of the Korean National Health Insurance Service-Health Screening (NHIS-HealS) Cohort data (2002–2003, with follow-up until 2019) can be found in another report [18,19].

### 2.2. Exposure (Chronic Kidney Disease)

Chronic kidney disease (CKD) was delineated based on participants receiving a diagnosis of CKD (ICD-10 codes: N18) on two or more occasions or being diagnosed with unspecified kidney failure (ICD-10 codes: N19). Furthermore, participants were considered eligible if they had undergone continuous hemodialysis, peritoneal dialysis, or both, as indicated by the respective procedure codes (O7010, O7020, and O7070).

### 2.3. Outcome (Chronic Rhinosinusitis)

Participants with chronic rhinosinusitis (CRS) were incorporated into the study if they had received a diagnosis of chronic sinusitis (ICD-10: J32). We chose participants who had undergone treatment multiple times and had undergone CT scan (medical claim codes: HA401 to HA416, HA441 to HA443, HA451 to HA453, HA461 to HA463, or HA471 to HA473). CRS was categorized into two groups: CRS with NPs and CRS without NPs, according to the treatment history for NPs (ICD-10: J33).

### 2.4. Selection of Participants

Individuals with CKD were identified from a pool of 514,866 individuals with a total of 895,300,177 claim codes spanning from 2002 through 2019, resulting in a final sample size of 17,478. The control group consisted of individuals who did not receive a diagnosis CKD from 2002 to 2019 (*n* = 497,388). To identify participants with a first-time diagnosis of CKD, individuals diagnosed with CKD in 2002 were removed (*n* = 536). CKD participants who did not have information for BMI, fasting blood sugar, and blood pressure were excluded (total, *n* = 5). Individuals in the control group who received a diagnosis with N18 (ICD-10 code) only once were removed (*n* = 560). Participants diagnosed with CKD were paired with control participants at a ratio of 1:4, matched according to age, sex, household income, and residential area. To mitigate selection bias in the selection of matched participants, control participants were arranged in random numerical order and subsequently chosen in a top-to-bottom sequence.

**Figure 1:**
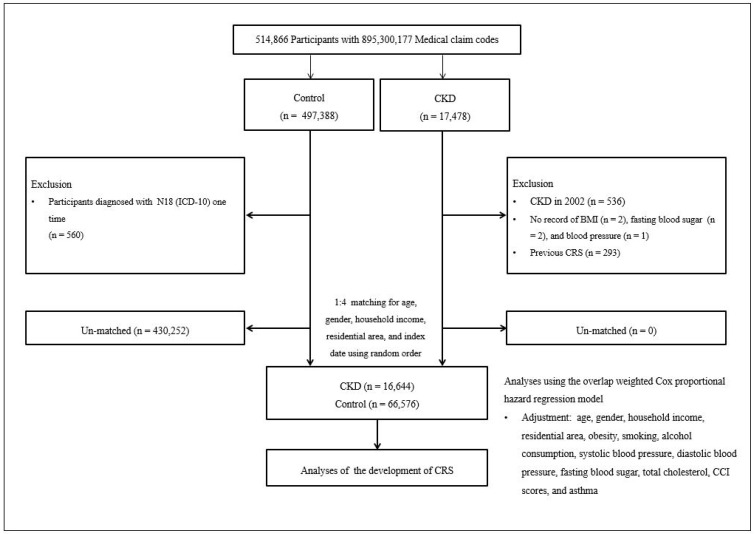
Diagram of the participant selection process. Out of a total of 514,866 participants, 16,644 with CKD were matched with 66,576 control participants based on age, gender, household income, and residential area.

It was presumed that the control participants, matched to the CKD participants, underwent evaluation concurrently with each corresponding CKD participant (index date). Consequently, individuals in the control group who were deceased prior to the index date were removed. Participants in both the CKD and control groups with preexisting information of CRS prior to the index date were also removed. Within the CKD group, 293 participants were removed due to left truncation. In the matching process, 430,252 control individuals were removed. Ultimately, 16,644 CKD participants were paired with 66,576 control individuals at a ratio of 1:4. (Figure 1).

### 2.5. Covariates

Age groups were defined in 5-year increments, spanning from 40–44 years old to 85 years and above, resulting in 10 distinct age categories overall. Income groups were stratified into 5 classes, with Class 1 indicating the lowermost income and Class 5 representing the uppermost income. The residential area was categorized into urban and rural regions, consistent with our prior report [20]. Cigarette smoking, alcohol ingestion, and obesity, assessed by BMI (body mass index, kg/m^2^) were classified in accordance with the methods employed in our prior report [21]. Records of blood pressure (systolic and diastolic, mmHg) and total cholesterol (mg/dL) were used.

The Charlson Comorbidity Index (CCI) is extensively employed for assessing disease burden, incorporating 17 concurrent illnesses. Each participant was assigned a score determined by the severity and quantity of illnesses, resulting in a continuous variable for CCI ranging from 0 (indicating no concurrent illnesses) to 29 (reflecting multiple concurrent illnesses) [22,23]. In our analysis, the inclusion of CKD (ICD-10 code: N18 and N19) was removed from the calculation of the CCI score.

Participants with asthma were defined as those receiving treatment for asthma (ICD-10 code: J45) or experiencing status asthmaticus (J46) on at least two occasions, using asthma-related medications such as inhaled corticosteroids (ICSs) alone or in combination with long-acting β2-agonists (LABAs), oral leukotriene antagonists (LTRAs), short-acting β2-agonists (SABAs), systemic LABAs, xanthine derivatives, or systemic corticosteroids. This methodology was adapted from a formerly validated report [24].

### 2.6. Statistical Analyses

We applied propensity score overlap weighting to address covariate balance and augment the effective sample size. The propensity score (PS) was derived through multivariable logistic regression, including all pertinent covariates. For the calculation of overlap weighting, CKD participants were weighted according to their PS probability, whereas control participants were weighted based on the probability of 1-PS. The resulting overlap weights range from 0 to 1, ensuring precise balance and optimization of precision [25,26,27]. The Standardized Difference, both before and after weighting, were employed to assess the disparities in general characteristics between the CKD and control groups (Table 1).

To examine the overlap-weighted hazard ratios (HRs) of CKD for any CRS, CRS with NPs, and CRS without NPs, we employed a propensity score overlap-weighted Cox proportional hazard regression model. In these examinations, both crude and overlap-weighted models were utilized. The overlap-weighted model was adjusted for various factors, including age, gender, household income, residential area, obesity, cigarette smoking, alcohol ingestion, systolic blood pressure (SBP), diastolic blood pressure (DBP), fasting blood sugar, total cholesterol, CCI scores, and asthma. The results are presented in Table 2, Table 3 and Table 4.

**Figure 2:**
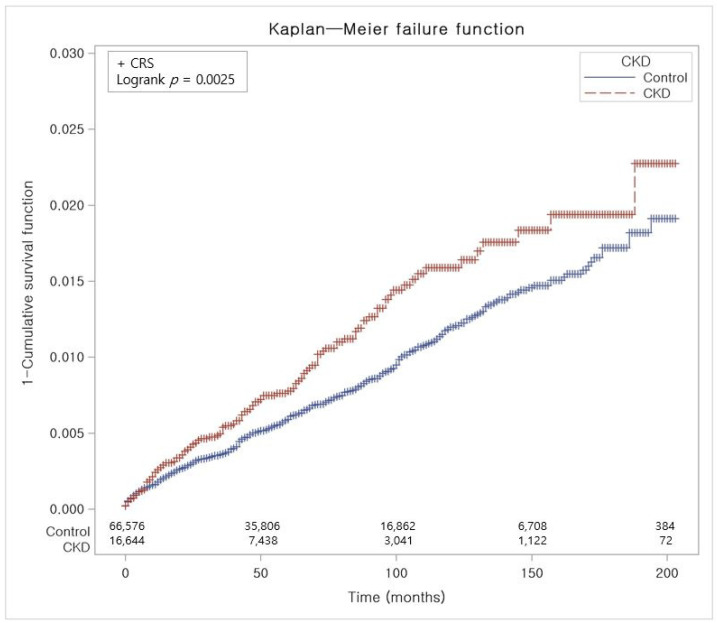
The risk of developing CRS in participants with and without CKD. Abbreviation: CRS, chronic rhinosinusitis; CKD, chronic kidney disease.

**Figure 3:**
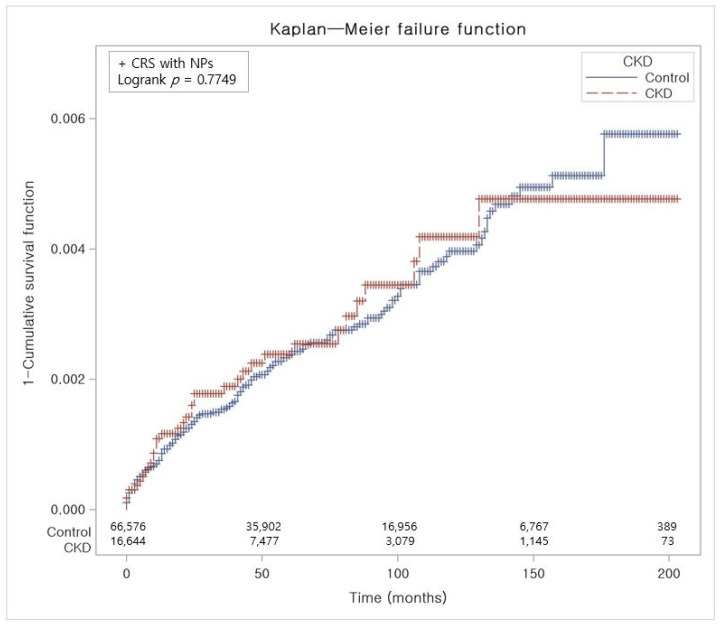
The risk of developing CRS with NPs in participants with and without CKD. Abbreviation: CRS, chronic rhinosinusitis; NP, nasal polyp; CKD, chronic kidney disease.

**Figure 4:**
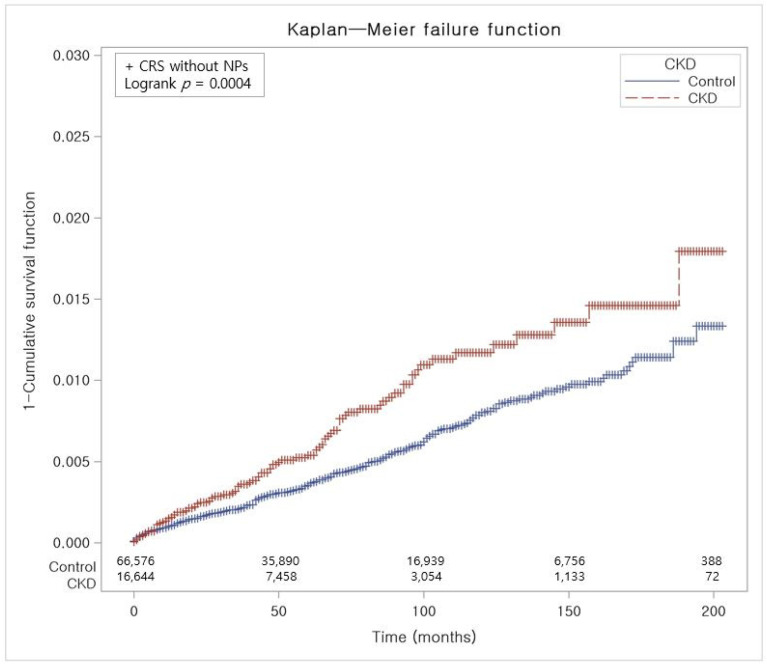
The risk of developing CRS without NPs in participants with and without CKD. Abbreviation: CRS, chronic rhinosinusitis; NP, nasal polyp; CKD, chronic kidney disease.

The crude incidence rate (IR) and incidence rate difference (IRD) were determined by dividing the number of individuals experiencing a specific occurrence by person-years, expressed as cases per 10,000 person-years. The Kaplan–Meier (KM) method was applied to compare the occurrence of any CRS, CRS with NPs, and CRS without NPs between the CKD participants and the control group, utilizing log-rank tests (Figure 2, Figure 3 and Figure 4). Two-tailed tests were performed, and statistical significance was determined with *p* values less than 0.05. Statistical investigations were performed utilizing SAS version 9.4 (SAS Institute Inc., Cary, NC, USA).

## 3. Results

For this study, we included a total of 16,644 individuals who were diagnosed with CKD, along with 66,576 controls matched based on relevant criteria. The characteristics of the participants are summarized in Table 1. Both groups showed a higher percentage of males and a larger proportion of non-smokers. Before the overlap adjustment, no significant disparities were observed in terms of age, gender, and residential area between the two groups. With the exception of CCI score and fasting blood sugar, most variables had standardized differences of 0.2 or less and demonstrated comparability between the CKD and control groups. The CCI score and fasting blood sugar standardized differences are 0.53 and 0.30, respectively, reflecting statistically significant differences, but after the overlap weighting adjustment, all of the variables’ standardized differences decreased to 0.00. This suggests that there are no significant discrepancies between the CKD and control groups after adjustment. The CRS was developed in 129 (0.29%) individuals in the CKD group and 453 (0.68%) individuals in the control groups.

Compared to the control group, which exhibited an IR of 13.10 per 10,000 person-years (IRD: HR 5.20, 95% CI: 2.16 to 8.22), the CKD group displayed a higher IR of CRS at 18.30 per 10,000 person-years. The CKD group showed a significantly higher incidence of CRS compared to the control group after adjusting for covariates (HR 1.28, 95% CI 1.09–1.5, *p* = 0.002) (Table 2).

In contrast to the control group, Kaplan–Meier analysis and a log-rank test showed a statistically notable elevation of cumulative incidence of CRS in the CKD group for the period of 17 years (Figure 2). The cumulative incidence of CRS with NPs in the CKD group did not exhibit a significant elevation in comparison to the control group (Figure 3). However, CRS without NPs in the CKD group demonstrated a significantly increased cumulative incidence over the 17-year period (Figure 4).

The subgroup analysis exposed variations in the risk of CRS among participants with CKD. After adjustment with overlap weighting, the CKD group exhibited a significantly higher HR for CRS in specific subgroups, including those under 70 years old, males, individuals in high-income households, and those residing in rural area (Table 2).

In our additional analysis, we examined the HR for CRS based on the presence of NPs. The CKD group did not exhibit a significantly higher HR for CRS with NPs (Table 3). However, within several subgroups (participants under 70 years old, male, individuals in high-income households, and those residing in rural areas), the CKD group demonstrated a significantly elevated HR for CRS without NPs (Table 4).

## 4. Discussion

This study demonstrated a significant relationship between CKD and CRS. Our findings suggest that patients with CKD have an advanced risk of emerging CRS than controls. This association persisted even after adjusting for demographic factors and comorbidities.

Only a few studies have considered the association between CKD and CRS, and the reports were controversial. Seo et al. reported the clinical characteristics of CRS [28], and they discovered that the prevalence of CRS in patients with end-stage renal disease (ESRD) was 2.5%. They recommended CRS screening because a significant number of patients were asymptomatic. They also demonstrated that the frequency of postoperative bleeding was much higher in patients with ESRD than in healthy patients with sinusitis. In contrast, a study about comorbidities in patients with CKD using nationally representative samples from Scotland [29] showed that the adjusted odds ratio (OR) for chronic sinusitis in patients with CKD was not statistically significant.

The underlying pathogenic mechanism for developing CRS in patients is still unclear; however, several possible explanations exist. One is prolonged mucociliary clearance time in the sinonasal mucosa. One study focused on nasal mucociliary clearance in patients undergoing peritoneal dialysis [30]. However, mucociliary clearance was not markedly prolonged in patients with CKD compared to healthy individuals. Another study demonstrated that the mucociliary clearance time was extended in patients undergoing hemodialysis and with chronic renal failure [31]. The mucociliary activity of the sinonasal mucosa (respiratory mucosa) is crucial because of its ability to remove foreign particles and pathogens associated with the development of rhinosinusitis. Although further studies are required, these findings suggest that mucociliary dysfunction in patients with CKD may play a role in the onset of CRS.

Another possible explanation is that CKD is closely associated with infection susceptibility. Chronic renal failure leads to an immunocompromised state accompanied by malnutrition and damage to the immune system [32,33]. Specifically, in patients with uremia, neutrophils demonstrate decreased bactericidal ability, and monocytes and macrophages show diminished antimicrobial capacity [34]. Additionally, naïve and memory T-cells exhibit an increased vulnerability to activation-triggered apoptosis [35,36]. In addition to changes and dysfunction in the immune system, other important factors contributing to immune vulnerability in CKD include gastrointestinal dysbiosis, oxidative stress, inflammation, and endocrine abnormalities. These abnormalities include alterations in parathyroid hormone and fibroblast growth factor 23 (FGF23) concentration, decreased vitamin D production, and changes in renin–angiotensin system function [34]. The effects of uremic conditions are associated with an increased susceptibility to infections, which may lead to the development of CRS.

While there was a positive correlation between CRS and CKD among all participants, certain subgroups, including those aged >70 years, females, individuals with low income, and urban residents, did not exhibit significant associations.

A recent systematic review of the risk factors for CRS in the Chinese population revealed that individuals residing in urban areas exhibited a lower prevalence of CRS [37]. However, other reports have focused on the impact of environmental factors, including air pollution, on CRS development [38]. Prolonged exposure to environmental air pollution in urban areas has been linked to chronic, localized, and systemic inflammation mediated by various pathways. These pathways include disruption of cilia in nasal mucosal epithelial cells, alterations in sinus bacterial colonization, promotion of bacterial biofilm and reactive oxygen species (ROS) formation, heightened secretion of pro-inflammatory cytokines, impairment of mucociliary clearance, epithelial barrier function, and immune balance [39,40,41,42,43,44,45,46,47,48,49,50]. A previous study indicated that male sex and aging were risk factors for both CRS and NP development [51]. Additionally, in a Korean national health dataset, a noteworthy association was observed between CRS with NPs and old age, and a lower level of education [52]. Lower levels of education may be linked to lower income, potentially resulting in delayed access to appropriate treatment for symptoms or relatively lower levels of hygiene.

Therefore, advanced age, low income, and residence in urban areas may be considered potential risk factors for the development of CRS. We assumed that the reason for the non-significant association in these subgroups was as follows: CKD can also serve as a risk factor for CRS, and there may be no significant differences in the development of CRS between individuals with CKD and controls. In addition, this study found no significant differences between the female groups. The HR for CRS was not statistically significant in the female group (*p* = 0.529), which could have been influenced by the relatively small number of female patients. Similarly, the number of older patients, those with low income, and urban residents with CKD was also relatively small, suggesting limitations in confirming the impact of these factors.

The current study had several limitations. First, participants were selected based on diagnostic codes. Even though many medical claims codes indicate CKD diagnoses, information on the exact blood urea nitrogen and creatinine levels may guarantee a significantly more accurate diagnosis. Hence, this study was unable to integrate disease severity. Since information on the serum levels of CKD markers guarantee an exact investigation, future analyses may be required to incorporate the serum levels of these markers. Second, CRS endotypes were not considered in this study. We classified the patients with CRS into groups with and without NP. However, a recent study showed that CRS can be categorized into five different groups of inflammatory cytokines [53]. These researchers proposed that CRS is not a strictly binary but rather a multidimensional disorder guided by varying inflammatory mechanisms. Additional studies incorporating CRS endotypes are necessitated to elucidate the association between CRS and CKD further.

Despite these limitations, the present research exhibits several strengths. First, we analyzed a substantial subset from a national cohort, ensuring study reliability. To our knowledge, this is the largest study to elucidate the association between CRS and CKD. Second, potential biases were well-controlled in the present study. Strict selection criteria for CKD and controls reduced selection bias. In addition, the participants were matched according to demographic factors, lifestyle factors, and laboratory data, and we adjusted for various other possible confounding variables, which incorporated not just CRS risk factors but also CKD, which might be mutually related.

## 5. Conclusions

This study showed that CKD was associated with augmented CRS risk. Additional analyses demonstrated that this effect was particularly enhanced in the participants with CRS without NPs. Further studies should thoroughly elucidate the connection between CKD and CRS and the underlying mechanisms.

## Figures and Tables

**Table 1:** General characteristics of participants.

Characteristics	Before Overlap Weighting Adjustment			After Overlap Weighting Adjustment		
	CKD	Control	Standardized Difference	CKD	Control	Standardized Difference
Age (*n*, %)			0.00			0.00
40–44	98 (0.59)	392 (0.59)		74 (0.60)	74 (0.60)	
45–49	358 (2.15)	1432 (2.15)		259 (2.10)	259 (2.10)	
50–54	937 (5.63)	3748 (5.63)		680 (5.51)	680 (5.51)	
55–59	1834 (11.02)	7336 (11.02)		1342 (10.87)	1342 (10.87)	
60–64	2267 (13.62)	9068 (13.62)		1661 (13.42)	1661 (13.42)	
65–69	2563 (15.40)	10,252 (15.40)		1882 (15.25)	1882 (15.25)	
70–74	2915 (17.51)	11,660 (17.51)		2167 (17.56)	2167 (17.56)	
75–79	2855 (17.15)	11,420 (17.15)		2146 (17.39)	2146 (17.39)	
80–84	1895 (11.39)	7580 (11.39)		1422 (11.52)	1422 (11.52)	
85+	922 (5.54)	3688 (5.54)		709 (5.75)	709 (5.75)	
Gender (*n*, %)			0.00			0.00
Male	10,915 (65.58)	43,660 (65.58)		8107 (65.68)	8107 (65.68)	
Female	5729 (34.42)	22,916 (34.42)		4237 (34.32)	4237 (34.32)	
Income (*n*, %)			0.00			0.00
1 (lowest)	2913 (17.50)	11,652 (17.50)		2150 (17.42)	2150 (17.42)	
2	1921 (11.54)	7684 (11.54)		1428 (11.57)	1428 (11.57)	
3	2362 (14.19)	9448 (14.19)		1747 (14.16)	1747 (14.16)	
4	3304 (19.85)	13,216 (19.85)		2444 (19.80)	2444 (19.80)	
5 (highest)	6144 (36.91)	24,576 (36.91)		4575 (37.06)	4575 (37.06)	
Residential area (*n*, %)			0.00			0.00
Urban	7136 (42.87)	28,544 (42.87)		5292 (42.87)	5292 (42.87)	
Rural	9508 (57.13)	38,032 (57.13)		7052 (57.13)	7052 (57.13)	
Obesity † (*n*, %)			0.16			0.00
Underweight	422 (2.66)	2287 (3.44)		348 (2.82)	348 (2.82)	
Normal	5125 (30.79)	23,567 (35.40)		3909 (31.67)	3909 (31.67)	
Overweight	4349 (26.13)	18,048 (27.11)		3263 (26.44)	3263 (26.44)	
Obese I	5969 (35.86)	20,836 (31.30)		4325 (35.04)	4325 (35.04)	
Obese II	759 (4.56)	1838 (2.76)		498 (4.04)	498 (4.04)	
Smoking status (*n*, %)			0.02			0.00
Nonsmoker	10,656 (64.02)	43,164 (64.83)		7933 (64.27)	7933 (64.27)	
Past smoker	1735 (10.42)	7073 (10.62)		1303 (10.56)	1303 (10.56)	
Current smoker	4253 (25.55)	16,339 (24.54)		3108 (25.18)	3108 (25.18)	
Alcohol consumption (*n*, %)			0.07			0.00
<1 time a week	12,109 (72.75)	46,468 (69.80)		8888 (72.00)	8888 (72.00)	
≥1 time a week	4535 (27.25)	20,108 (30.20)		3456 (28.00)	3456 (28.00)	
Systolic blood pressure (Mean, SD)	131.87 (18.39)	128.77 (16.33)	0.18	130.95 (15.44)	130.95 (7.35)	0.00
Diastolic blood pressure (Mean, SD)	78.75 (11.50)	78.11 (10.35)	0.06	78.54 (9.80)	78.54 (4.57)	0.00
Fasting blood sugar (Mean, SD)	115.63 (49.21)	103.65 (28.22)	0.30	109.99 (32.72)	109.99 (16.91)	0.00
Total cholesterol (Mean, SD)	190.37 (45.72)	193.63 (38.99)	0.08	190.87 (39.13)	190.87 (16.94)	0.00
CCI score (Mean, SD)	2.18 (2.20)	1.13 (1.73)	0.53	1.84 (1.68)	1.84 (0.98)	0.00
Asthma (*n*, %)	5341 (32.09)	20,485 (30.77)	0.03	3948 (31.98)	3948 (31.98)	0.00
Any CRS (*n*, %)	129 (0.78)	453 (0.68)	0.01	97 (0.78)	88 (0.71)	0.01
CRS with NPs	37 (0.22)	163 (0.24)	0.01	27 (0.22)	30 (0.25)	0.01
CRS without NPs	92 (0.55)	290 (0.44)	0.02	70 (0.56)	58 (0.47)	0.01

Abbreviations: CCI, Charlson Comorbidity Index; CKD, chronic kidney disease; CRS, chronic rhinosinusitis; NP, nasal polyp; † Obesity (BMI, body mass index, kg/m^2^) was classified as <18.5 (underweight), ≥18.5 to <23 (normal), ≥23 to <25 (overweight), ≥25 to <30 (obese I), and ≥30 (obese II).

**Table 2:** Crude and overlap propensity score weighted hazard ratios (95% confidence interval) of CKD for CRS with subdivision analyses according to age, gender, household income, and residential area.

Characteristics					Hazard Ratios for CRS			
	*n* of Event /*n* of Total (%)	Follow-Up Duration (PY)	IR per 10,000 (PY)	IRD (95% CI)	Crude	*p*-Value	Overlap Weighted Model †	*p*-Value
Total participants								
CKD	129/16,644 (0.78)	70,439	18.30	5.20 (2.16 to 8.22)	1.35 (1.11–1.64)	0.003 *	1.28 (1.09–1.5)	0.002 *
Control	453/66,576 (0.68)	345,144	13.10		1		1	
Age < 70 years old								
CKD	91/8057 (1.13)	45,126	20.20	4.80 (0.65 to 8.83)	1.28 (1.01–1.61)	0.039 *	1.26 (1.04–1.51)	0.016 *
Control	330/32,228 (1.02)	213,949	15.40		1		1	
Age ≥ 70 years old								
CKD	38/8587 (0.44)	25,313	15.00	5.62 (1.32 to 9.95)	1.49 (1.03–2.14)	0.033 *	1.33 (0.99–1.79)	0.06
Control	123/34,348 (0.36)	131,195	9.38				1	
Male								
CKD	90/10,915 (0.82)	45,143	19.90	6.50 (2.66 to 10.38)	1.44 (1.13–1.82)	0.003 *	1.36 (1.12–1.65)	0.002 *
Control	297/43,660 (0.68)	221,354	13.40				1	
Female								
CKD	39/5729 (0.68)	25,296	15.40	2.80 (−2.08 to 7.71)	1.19 (0.84–1.69)	0.332	1.09 (0.83–1.45)	0.529
Control	156/22,916 (0.68)	123,790	12.60		1		1	
Low income								
CKD	54/7196 (0.75)	30,013	18.00	5.60 (1.06 to 10.12)	1.41 (1.04–1.90)	0.028 *	1.22 (0.96–1.56)	0.101
Control	186/28,784 (0.65)	149,978	12.40		1		1	
High income								
CKD	75/9448 (0.79)	40,426	18.60	4.90 (0.79 to 8.95)	1.31 (1.02–1.70)	0.037 *	1.31 (1.07–1.62)	0.01 *
Control	267/37,792 (0.71)	195,166	13.70		1		1	
Urban resident								
CKD	56/7136 (0.78)	32,128	17.40	4.40 (−0.03 to 8.89)	1.31 (0.97–1.76)	0.078	1.21 (0.96–1.53)	0.108
Control	200/28,544 (0.70)	153,845	13.00		1		1	
Rural resident								
CKD	73/9508 (0.77)	38,311	19.10	5.90 (1.70 to 9.96)	1.39 (1.07–1.80)	0.013 *	1.33 (1.08–1.65)	0.008 *
Control	253/38,032 (0.67)	191,299	13.20		1		1	

Abbreviations: IR, incidence rate; IRD, incidence rate difference; PY, person-year * Significance at *p* < 0.05. † Adjusted for age, gender, household income, residential area, obesity, smoking, alcohol consumption, systolic blood pressure, diastolic blood pressure, fasting blood sugar, total cholesterol, CCI scores, and asthma).

**Table 3:** Crude and overlap propensity score weighted hazard ratios (95% confidence interval) of CKD for the CRS with NPs subgroup analyses according to age, gender, household income, and residential area.

Characteristics					Hazard Ratios for CRS			
	*n* of Event /*n* of Total (%)	Follow-Up Duration (PY)	IR per 10,000 (PY)	IRD (95% CI)	Crude	*p*-Value	Overlap Weighted Model †	*p*-Value
Total participants								
CKD	37/16,644 (0.22)	70,876	5.22	0.51 (−1.26 to 2.28)	1.05 (0.74–1.51)	0.775	1.01 (0.76–1.34)	0.946
Control	163/66,576 (0.24)	346,290	4.71		1		1	
Age < 70 years old								
CKD	24/8057 (1.13)	45,477	5.28	−0.54 (−2.96 to 1.88)	0.87 (0.56–1.35)	0.532	0.90 (0.64–1.26)	0.521
Control	125/32,228 (1.02)	214,869	5.82		1		1	
Age ≥ 70 years old								
CKD	13/8587 (0.15)	25,399	5.12	2.23 (−0.20 to 4.65)	1.61 (0.86–3.02)	0.14	1.42 (0.84–2.40)	0.196
Control	38/34,348 (0.11)	131,421	2.89				1	
Male								
CKD	25/10,915 (0.23)	45,462	5.50	0.23 (−2.10 to 2.55)	0.99 (0.64–1.53)	0.972	0.97 (0.69–1.36)	0.844
Control	117/43,660 (0.27)	222,014	5.27				1	
Female								
CKD	12/5729 (0.21)	25,414	4.72	−0.31 (−0.63 to 0.01)	1.21 (0.64–2.29)	0.554	1.10 (0.65–1.84)	0.725
Control	46/22,916 (0.20)	124,276	3.70		1		1	
Low income								
CKD	17/7196 (0.24)	30,202	5.63	1.38 (−1.24 to 3.99)	1.24 (0.73–2.12)	0.427	1.03 (0.67–1.57)	0.904
Control	64/28,784 (0.22)	150,474	4.25		1		1	
High income								
CKD	20/9448 (0.21)	40,674	4.92	−0.14 (−2.53 to 2.26)	0.93 (0.58–1.51)	0.771	0.99 (0.67–1.45)	0.949
Control	99/37,792 (0.26)	195,816	5.06		1		1	
Urban resident								
CKD	19/7136 (0.27)	32,274	5.89	1.55 (−1.03 to 4.12)	1.31 (0.78–2.16)	0.315	1.28 (0.85–1.95)	0.24
Control	67/28,544 (0.23)	154,406	4.34		1		1	
Rural resident								
CKD	18/9508 (0.19)	38,602	4.66	−0.34 (−2.77 to 2.09)	0.88 (0.53–1.46)	0.619	0.81 (0.55–1.20)	0.291
Control	96/38,032 (0.25)	191,884	5.00		1		1	

Abbreviations: IR, incidence rate; IRD, incidence rate difference; PY, person-year. † Adjusted for age, gender, household income, residential area, obesity, smoking, alcohol consumption, systolic blood pressure, diastolic blood pressure, fasting blood sugar, total cholesterol, CCI scores, and asthma).

**Table 4:** Crude and overlap propensity score weighted hazard ratios (95% confidence interval) of CKD for the CRS without NPs subgroup analyses according to age, gender, household income, and residential area.

Characteristics					Hazard Ratios for CRS			
	*n* of Event /*n* of Total (%)	Follow-up Duration (PY)	IR per 10,000 (PY)	IRD (95% CI)	Crude	*p*-Value	Overlap Weighted Model †	*p*-Value
Total participants								
CKD	92/16,644 (0.55)	70,652	13.00	4.62 (2.19 to 7.09)	1.52 (1.20–1.92)	<0.001 *	1.42 (1.17–1.72)	<0.001 *
Control	290/66,576 (0.44)	346,118	8.38		1		1	
Age <70 years old								
CKD	67/8057 (0.83)	45,294	14.80	5.26 (1.97 to 8.52)	1.53 (1.16–2.02)	0.003 *	1.46 (1.16–1.82)	0.001 *
Control	205/32,228 (0.64)	214,778	9.54		1		1	
Age ≥70 years old								
CKD	25/8587 (0.29)	25,358	9.86	3.39 (−0.17 to 6.95)	1.43 (0.91–2.23)	0.119	1.29 (0.90–1.85)	0.169
Control	85/34,348 (0.25)	131,340	6.47				1	
Male								
CKD	65/10,915 (0.60)	45,273	14.40	6.29 (3.19 to 9.31)	1.73 (1.30–2.30)	<0.001 *	1.59 (1.26–2.01)	<0.001 *
Control	180/43,660 (0.41)	222,032	8.11				1	
Female								
CKD	27/5729 (0.47)	25,379	10.60	1.74 (−2.31 to 5.86)	1.18 (0.77–1.80)	0.444	1.09 (0.78–1.53)	0.615
Control	110/22,916 (0.48)	124,086	8.86		1		1	
Low income								
CKD	37/7196 (0.51)	30,138	12.30	4.19 (0.49 to 7.84)	1.49 (1.03–2.16)	0.033 *	1.33 (0.99–1.78)	0.059
Control	122/28,784 (0.42)	150,376	8.11		1		1	
High income								
CKD	55/9448 (0.58)	40,514	13.60	5.02 (1.71 to 8.28)	1.54 (1.14–2.09)	0.005 *	1.49 (1.16–1.91)	0.002 *
Control	168/37,792 (0.44)	195,742	8.58		1		1	
Urban resident								
CKD	37/7136 (0.52)	32,233	11.50	2.88 (−0.77 to 6.48)	1.31 (0.91–1.88)	0.149	1.18 (0.89–1.57)	0.25
Control	133/28,544 (0.47)	154,246	8.62		1		1	
Rural resident								
CKD	55/9508 (0.58)	38,419	14.30	6.12 (2.81 to 9.46)	1.71 (1.26–2.32)	<0.001 *	1.66 (1.28–2.14)	<0.001 *
Control	157/38,032 (0.41)	191,872	8.18		1		1	

Abbreviations: IR, incidence rate; IRD, incidence rate difference; PY, person-year * Significance at *p* < 0.05. † Adjusted for age, gender, household income, residential area, obesity, smoking, alcohol consumption, systolic blood pressure, diastolic blood pressure, fasting blood sugar, total cholesterol, CCI scores, and asthma).

## Data Availability

These data are subject to certain limitations regarding their availability. They were acquired from the Health Insurance Review and Assessment Service (HIRA) of Korea and can be accessed at https://opendata.hira.or.kr (accessed on 20 September 2023) with the authorization of HIRA.
